# Myeloid neoplasms with *MYC*-positive double minutes: towards recognition as a distinct entity

**DOI:** 10.1038/s41408-025-01244-6

**Published:** 2025-03-03

**Authors:** Isolde Summerer, Wencke Walter, Manja Meggendorfer, Torsten Haferlach, Frank Dicker, Stephan Hutter, Niroshan Nadarajah, Anna Stengel, Constanze Kuehn, Wolfgang Kern, John M. Bennett, Claudia Haferlach, Christian Pohlkamp

**Affiliations:** 1 MLL Munich Leukemia Laboratory, Munich, Germany; 2 University of Rochester Medical Center, Hematopathology Division, Department of Pathology, Rochester, NY USA

**Keywords:** Leukaemia, Disease genetics

**Dear Editor**,

In hematologic malignancies, the occurrence of double minutes (dmin) is a rare cytogenetic phenomenon, predominantly associated with acute myeloid leukemia (AML). The acentric chromosomal structures usually represent oncogene amplification, such as the *MYC* gene (Fig. 1A), highlighting a pivotal aspect of cancer genetics [1, 2]. Single case reports have indicated an association of *MYC*-positive dmin (*MYC* dmin) and cytomorphologic features similar to those observed in acute promyelocytic leukemia (APL) [3–6], including atypically granulated/hypergranulated promyelocytes with high numbers of Auer rods and even faggot cells [7]. However, comprehensive studies are missing so far. Our study aims to fill the gap in understanding the genotypical and phenotypical characteristics of myeloid neoplasms (MN) with *MYC* dmin. We retrospectively reviewed all cases with MN sent to the MLL Munich Leukemia Laboratory between November 2005 and November 2022, where chromosome banding analysis (CBA) was available. Of these 75,839 cases 76 harbored *MYC* dmin. We conducted a comprehensive characterization of these patients focusing on their cytomorphologic, cytogenetic, mutational, transcriptional and clinical features.

**Fig. 1:**
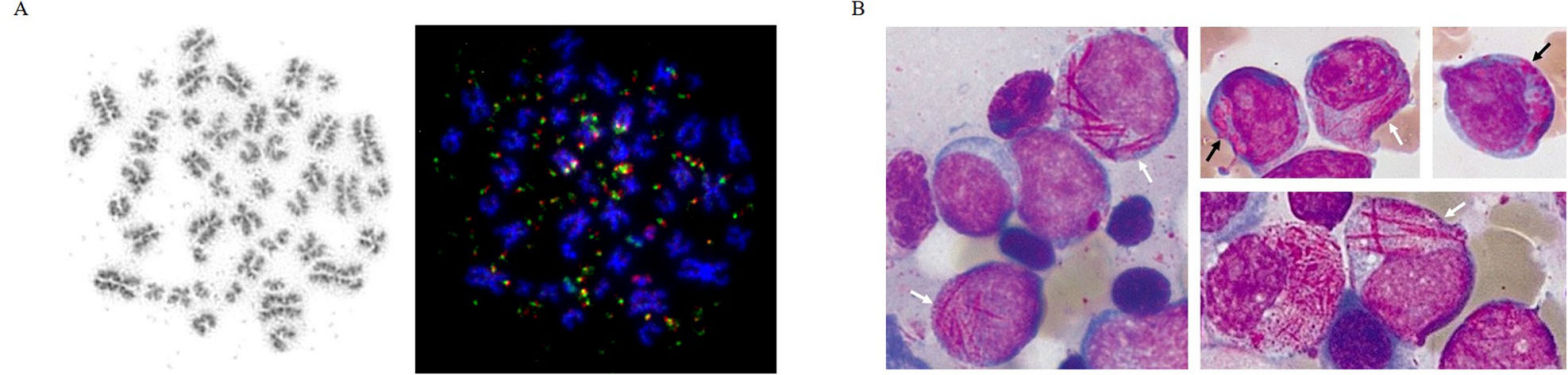
Characterization of *MYC*-positive double minutes (*MYC* dmin) cases of myeloid neoplasms with respect to cytogenetics and cytomorphology. **A** *MYC* dmin in chromosome banding analysis (left) and in metaphase-FISH analysis (right) using a dual color probe labeling the *MYC* gene (Chr 8q24.21) (MetaSystems XL MYC BA). **B** Typical cytomorphology in *MYC* dmin cases. Faggot cells with Auer rods (white arrows) and cells with pseudo Chediak-Higashi granules (black arrows).

Of the 76 patient samples (36 female, 40 male, median age 75 years (range 44–89 years)) (patient details in Supplementary Table 1) 60 were bone marrow (BM) and 16 peripheral blood (PB) samples. The diagnoses were established following WHO-HAEM5 guidelines [7]. All patients had given written informed consent to the use of genetic and clinical data according to the Declaration of Helsinki. Dmin and *MYC* amplification were assessed by CBA and fluorescence in situ hybridization (FISH) as previously described according to standard methods [8]. Cytomorphologic examination included retrospective assessment of APL-like features, i.e. high number of atypical hypergranulated promyelocytes, high number of Auer rods, faggot(-like) cells. We conducted comprehensive genomic analyses using a targeted next-generation sequencing panel of 72 myeloid genes across all patients, achieving a median coverage of 1500x. Whole-genome sequencing (WGS) (median coverage 100x) [9] was performed for a subgroup of 16 patients and whole-transcriptome sequencing (WTS) (50 million reads) [9] for 40 patients, where the required sample material was available, utilizing NovaSeq sequencing platforms. For gene expression (GE) analysis estimated read counts for each gene were normalized applying Trimmed mean of M-values normalization method and the resulting log2 counts per million (CPM) were used. Genes were kept if they were expressed (>5 CPM) in at least 66% of all samples. Variant calling for panel sequencing was performed using the Pisces software, with a minimum sensitivity threshold of 3%. We further compared mutation frequencies and GE patterns of *MYC* dmin cases with AML cases without *MYC* dmin (cohort details in Supplementary Fig. 1A), referred to as “non dmin” AML. Survival data were available for a subset of 49 patients (37 AML (32 AML-MR), 3 MDS with biallelic *TP53* inactivation, 4 CMML-2, 3 MDS/MPN, 1 MDS-IB1, 1 MPN in blast phase), with a median follow-up period of 18 months. Data on therapeutic treatment were available for 48 patients, of these, 19 received intensive therapy (Supplementary Table 1).

The majority of *MYC* dmin cases (55/76, 72%) were classified as AML-MR (myelodysplasia-related) according to WHO-HAEM5. Three (4%) cases each were classified as AML with maturation or without maturation, while one case with AML was not further classifiable due to insufficient sample quality and lack of defining markers. Further diagnoses were MDS with biallelic *TP53* inactivation (4/76, 5%), MDS-IB1 (1/76, 1%), CMML-2 (4/76, 5%), CMML-1 (1/76, 1%), MDS/MPN (3/76, 4%) or MPN in blast phase (1/76, 1%) (Supplementary Fig. 2). BM blast count was ≥10% in 52/60 (87%) patients, likely underestimated in the remaining 8 samples due to lack of particles. All 16 patients where only PB was available showed >2% blasts, 12 of these ≥20%, thus diagnosed as AML. Two of the PB cases were diagnosed as CMML-2, while one case each was diagnosed as MDS-IB1 or MDS/MPN, potentially underestimating the blast count due to the lack of BM. A significant finding was the high prevalence of dysplastic granulopoiesis across the cohort. 48 of the 50 patients (96%) where cytomorphology was fully assessable (Supplementary Fig. 1B) presented with a highly dysplastic granulopoiesis, often including severe dysplasia in other myeloid lineages, even in the absence of myelodysplasia-related genetic markers. APL-like features were detected in 48% of fully analyzable cases (24/50), albeit with a higher degree of neutrophilic maturation compared to APL with *PML*::*RARA* fusion (Fig. 1B). Moreover, pseudo Chediak-Higashi granules were often found in these cases (Fig. 1B).

Complex karyotypes (≥3 aberrations in addition to *MYC* dmin) were present in a significant portion of *MYC* dmin patients (25/76 patients (33%), whereas 21/76 (28%) patients presented with *MYC* dmin as the only cytogenetic aberration. Most frequent chromosomal gains were trisomy 4 (12/76, 16%), trisomy 6 (9/76, 12%) and trisomy 22 (8/76, 11%), while most frequent losses were del(5q) (22/76, 29%), del(17p) (19/76, 25%) and del(9q)/monosomy 9 (17/76, 22%) (Supplementary Fig. 3).

*TET2* mutations were the most frequent mutations in *MYC* dmin patients (55/76, 72%) (Fig. 2A), often biallelic or multi-hit (36/55, 65%) (27 cases >1 *TET2* mutation, 5 cases *TET2* mutation with VAF > 80%, 2 cases *TET2* mutation and deletion, 2 cases CN-LOH) (Fig. 2B). Also, *TET2* mutations were significantly overrepresented compared to “non dmin” AML [10] (72% vs. 19%, *p* < 0.001) (Fig. 2A) and often accompanied by trisomy 4 (Fig. 2B). *TET2* and *TP53* mutations (24/75, 32%) were almost mutually exclusive and only co-occured in six patients. *TP53* mutations showed a strong association with complex karyotypes (84% vs. 6% in non-complex karyotype, *p* < 0.001) (Fig. 2B). Except for two patients, *TP53* mutations and *U2AF1* mutations (20/75, 27%) were mutually exclusive. Interestingly, both mutations were highly overrepresented in *MYC* dmin patients compared to “non dmin” AML (*TP53*: 32% vs. 11%, *p* < 0.001; *U2AF1*: 27% vs. 4%, *p* < 0.001), whereas there was a complete absence of mutations in *NPM1, CEBPA, FLT3* or *WT1* in *MYC* dmin patients (Fig. 2A).

**Fig. 2:**
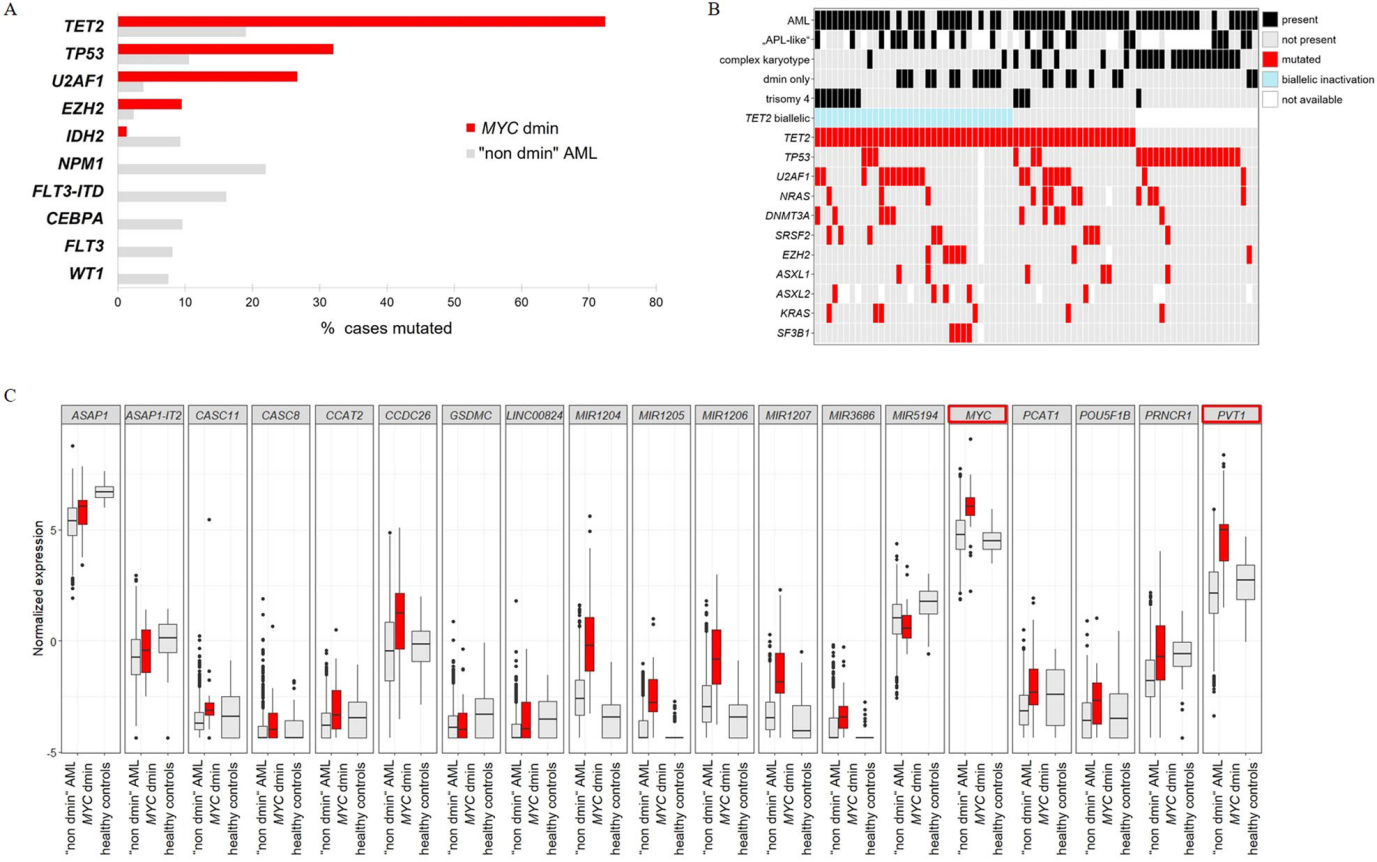
Genetics of *MYC*-positive double minutes (*MYC* dmin) cases. **A** Genes showing significantly different mutation frequencies in 76 patients with *MYC* dmin compared to 707 AML without *MYC*-positive double minutes (“non dmin” AML). **B** Oncoplot of 76 *MYC* dmin cases depicting diagnosis (AML or non-AML), presence of APL-like features, complex karyotype (≥3 chromosomal aberrations in addition to dmin), dmin only (no further chromosomal aberrations), the mutational status (for genes mutated in ≥4 patients), the presence of trisomy 4 and a biallelic inactivation status of *TET2*. **C** Box plots of normalized expression of genes located in the amplified chromosomal region of 40 *MYC* dmin cases (red) compared to 830 “non dmin” AML and 64 healthy controls. Differential expression was determined with the R package limma and values were corrected for multiple testing. Significantly overexpressed genes in *MYC* dmin cases are highlighted in red.

WGS identified an amplified chromosomal region with a size varying from 4.3 to 5.6 Mb and a commonly amplified region of 4.3 Mb (Chr 8:126,422,001–130,697,000) (Supplementary Fig. 4). This region encompasses 6 protein coding genes *ASAP1*, *CYRIB*, *GSMDC*, *LRATD2*, *POU5F1B* and *MYC* as well as several non-coding RNAs including the long non-coding RNA *PVT1* (Supplementary Table 2). An effect of the amplified region on GE was confirmed by overexpression of *MYC* (*p* < 0.001) and *PVT1* (*p* < 0.001) compared to “non dmin” AML [11] (Fig. 2C). A tSNE plot based on the top 5% of most variable genes of 40 *MYC* dmin cases analyzed by WTS demonstrated similar GE profiles within the *MYC* dmin cohort compared to “non dmin” AML cases (Supplementary Fig. 5).

The median overall survival of 49 evaluable *MYC* dmin patients was 11 months. Survival analysis revealed *TP53* mutations (Cox regression: HR: 5.1, *p* < 0.001), *NRAS* mutations (Cox regression: HR: 2.7, *p* = 0.036) and complex karyotype (Cox regression: HR: 2.6, *p* = 0.021) as associated with inferior survival within *MYC* dmin cases in a univariate analysis, whereas treatment with intensive therapy (Cox regression: HR: 0.3, *p* = 0.019) and *TET2* mutations (Cox regression: HR: 0.2, *p* < 0.001) were associated with better overall survival. Because of the strong negative association between *TET2* and *TP53* mutations, we performed a multivariate analysis including only *TP53* and *NRAS* mutations, complex karyotype and type of therapy. *TP53* (Cox regression: HR: 6.8, *p* = 0.005) and *NRAS* mutations (Cox regression: HR: 3.8, *p* = 0.011) emerged as independent predictors of inferior overall survival (Supplementary Fig. 6).

A more detailed analysis of the 24 cases showing the typical APL-like cytomorphology revealed a strong association with *U2AF1* mutations (50% vs. 15% in other *MYC* dmin cases, *p* = 0.004). Conversely, *TP53* mutations (13% vs. 40% in other *MYC* dmin cases, *p* = 0.017) and complex karyotypes very rarely (17% vs. 40% in other *MYC* dmin cases, n.s.) occurred in APL-like cases, which hints to a critical role of *MYC* dmin in developing the typical APL-like cytomorphology, while the accumulation of additional (cyto-)genetic alterations seem to dilute these phenotypic characteristics.

The detection of dmin in CBA is challenging due to their minuscule size and FISH analysis is usually performed to confirm the associated gene amplification. We show that MN with *MYC* dmin exhibit distinctive clinical and molecular features, often presenting with APL-like cytomorphology, especially in cases with *MYC* dmin as the predominant cytogenetic alteration. MN with *MYC* dmin are characterized by a high frequency of mutations in *TET2*, *TP53* and *U2AF1*, as well as relatively uniform GE profiles affected by a commonly amplified chromosomal region. The cytogenetic co-aberrations and the mutation patterns enable further differentiation within the cohort of *MYC* dmin cases. Particularly, in cases where APL is suspected but *PML*::*RARA* or other *RARA* fusions are excluded, CBA and FISH for the detection of *MYC* dmin represent valuable tools for identifying this distinct subtype, which is associated with a high blast count and severe dysplasia. Based on this in-depth characterization of to our knowledge the largest cohort of these rare cases, we propose that MN with *MYC* dmin might be considered a distinct genetically defined entity or at least, cases with acute leukemia and *MYC* dmin might be considered as such, possibly as a distinct subtype of AML-MR. Nonetheless, further research is needed for validation of our observations.

## Supplementary information

Supplementary Material

Supplementary Table 1

Supplementary Table 2

## Data Availability

The datasets generated during and/or analyzed during the current study are available from the corresponding author on reasonable request.
