# Supplementary material for: Myeloid neoplasms with MYC-positive double minutes: towards recognition as a distinct entity
Source: Blood Cancer J. 2025 Mar 3;15(1):29. doi: 10.1038/s41408-025-01244-6 (PMC11876648; doi:10.1038/s41408-025-01244-6)
Supplement: Supplementary file 1 — Supplementary Material [file 41408_2025_1244_MOESM1_ESM.pdf]

<u>**Supplementary Material**</u>

Supplementary Figures

A

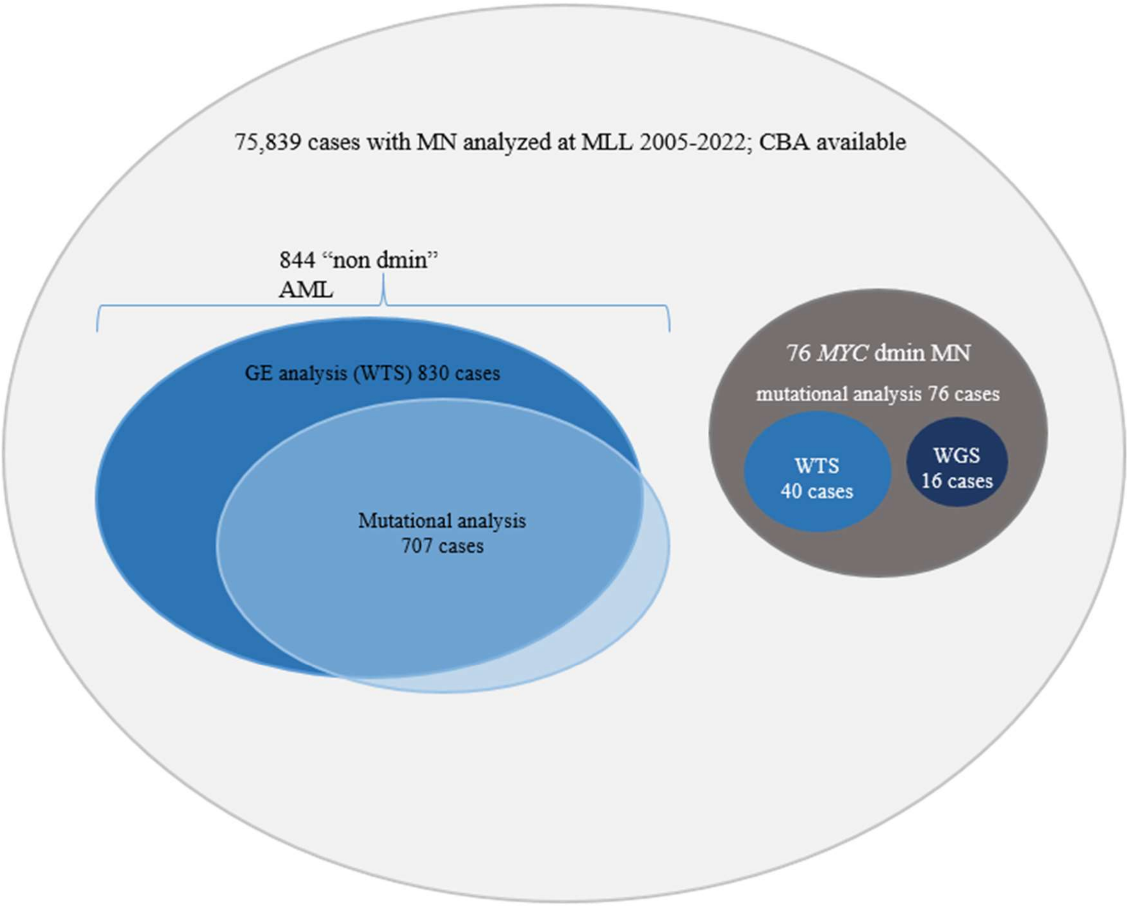

B

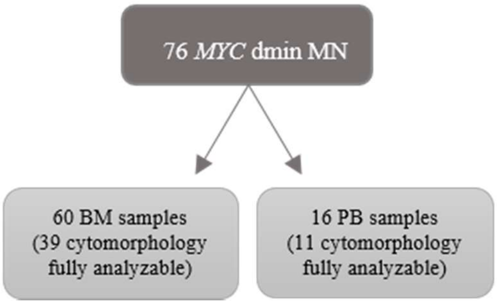

Supplementary Figure 1: (A) Overview of cohorts and analyses. The 707 AML cases used for comparison of somatic mutations of dmin *MYC* cases and "non dmin" AML were published in a previous study (Stengel A, Meggendorfer M, Walter W, et al. Interplay of TP53 allelic

state, blast count, and complex karyotype on survival of patients with AML and MDS. *Blood Adv*. 2023;7(18):5540-5548.). Gene expression profiles of *MYC* dmin cases were compared to 830 “non dmin” AML comprising 693 cases from the above mentioned study and 137 additional “non dmin” AML, which were analyzed by WTS in routine diagnostics. CBA: chromosome banding analysis, GE: gene expression, MLL: Munich Leukemia Laboratory, MN: myeloid neoplasms, WGS: whole genome sequencing, WTS: whole transcriptome sequencing **(B)** Sample distribution of *MYC* dmin cases and assessability of cytomorphology. BM: bone marrow, GE: gene expression, MN: myeloid neoplasms, PB: peripheral blood.

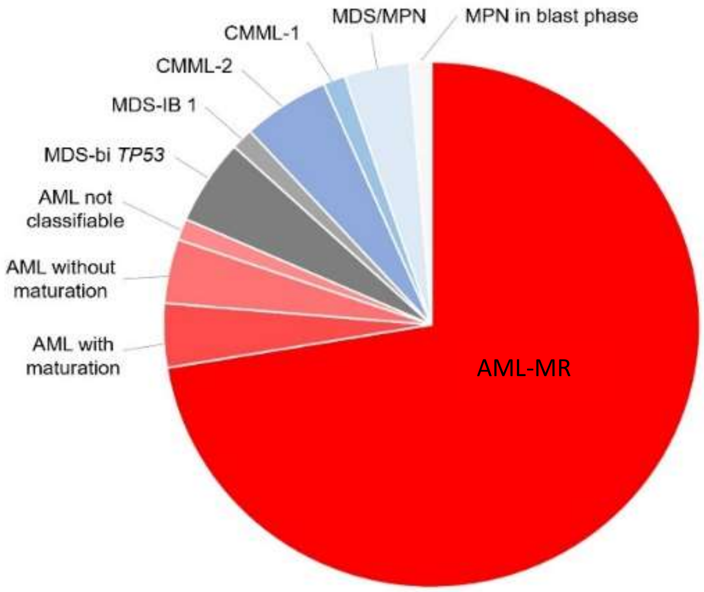

Supplementary Figure 2: Diagnoses of 76 *MYC* dmin cases according to WHO-HAEM5.

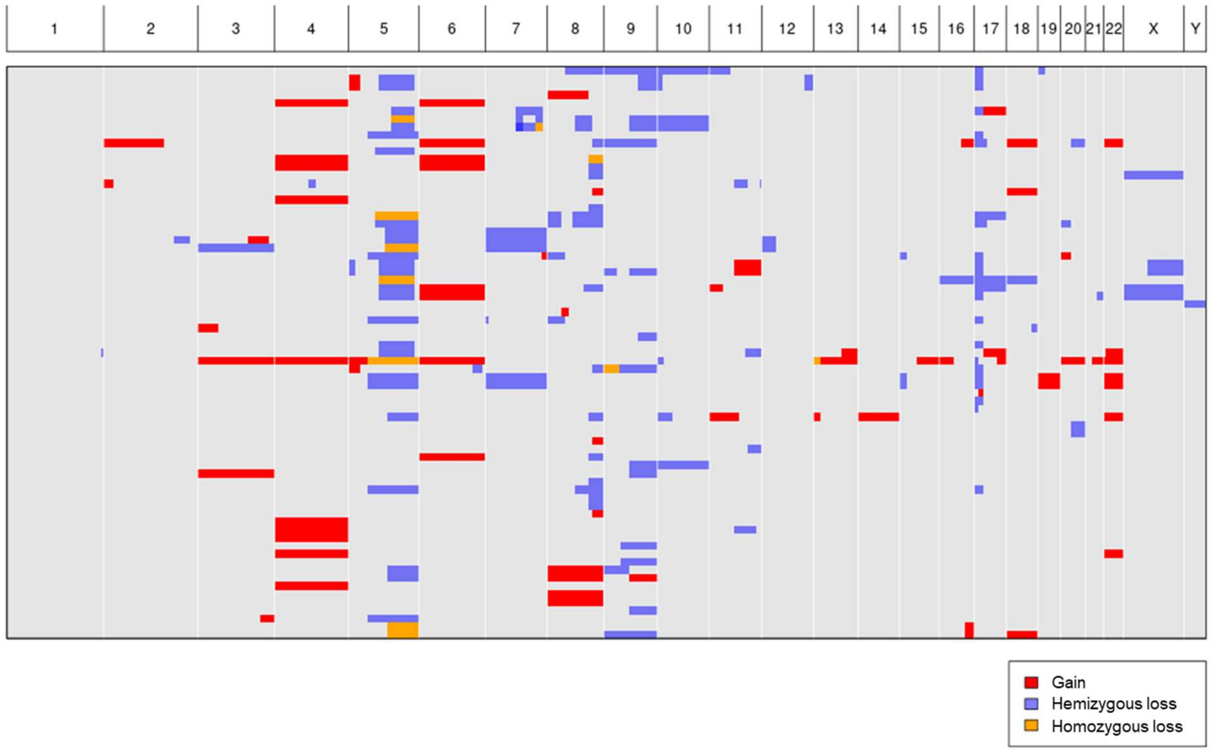

Supplementary Figure 3: Chromosomal losses (hemizygous: blue, homozygous: orange) and gains (red) in addition to *MYC* dmin of 76 patients based on chromosome banding analysis. Each line represents one patient. Chromosomes are outlined above. The plot was created with the web-based tool CytoConverter (Cytogenetic Nomenclature to Genomic Coordinate Translator: https://jxw773.shinyapps.io/Cytogenetic__software/).

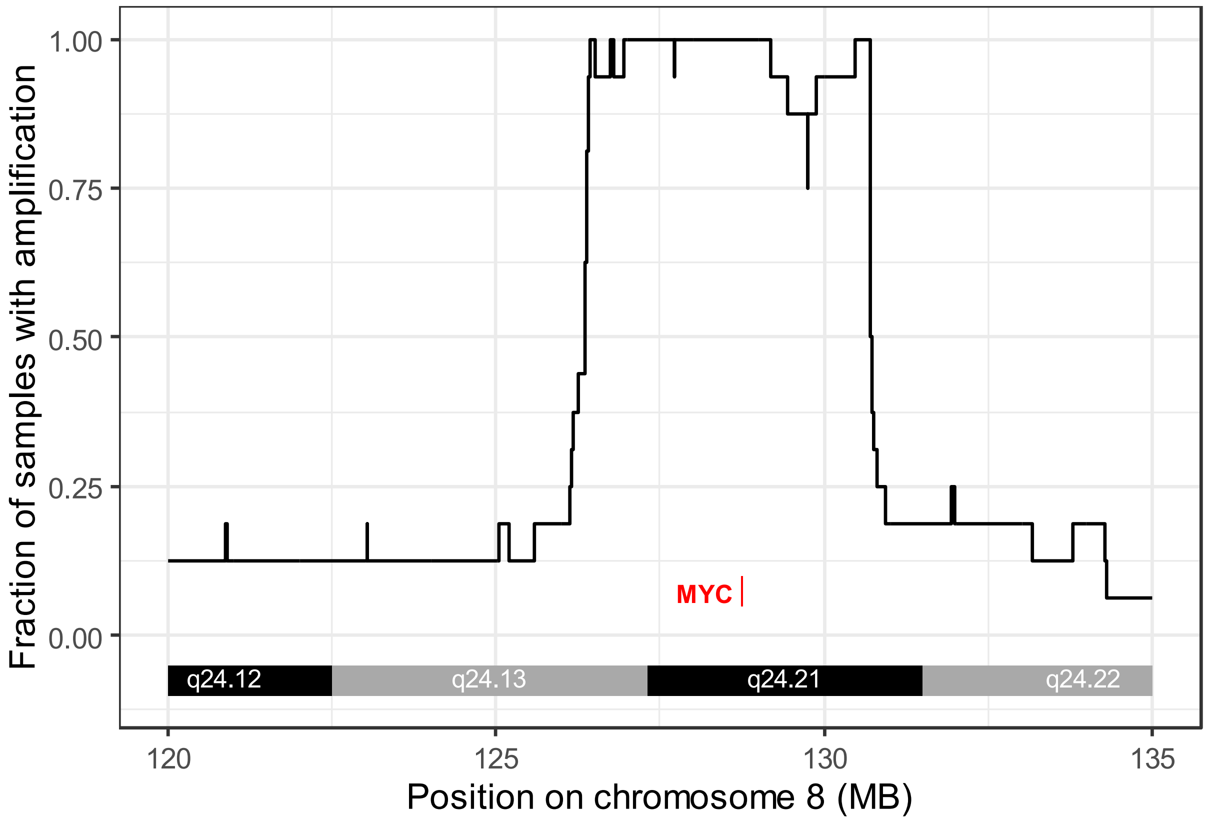

Supplementary Figure 4: Amplified region of 16 *MYC* dmin cases analyzed by whole genome sequencing.

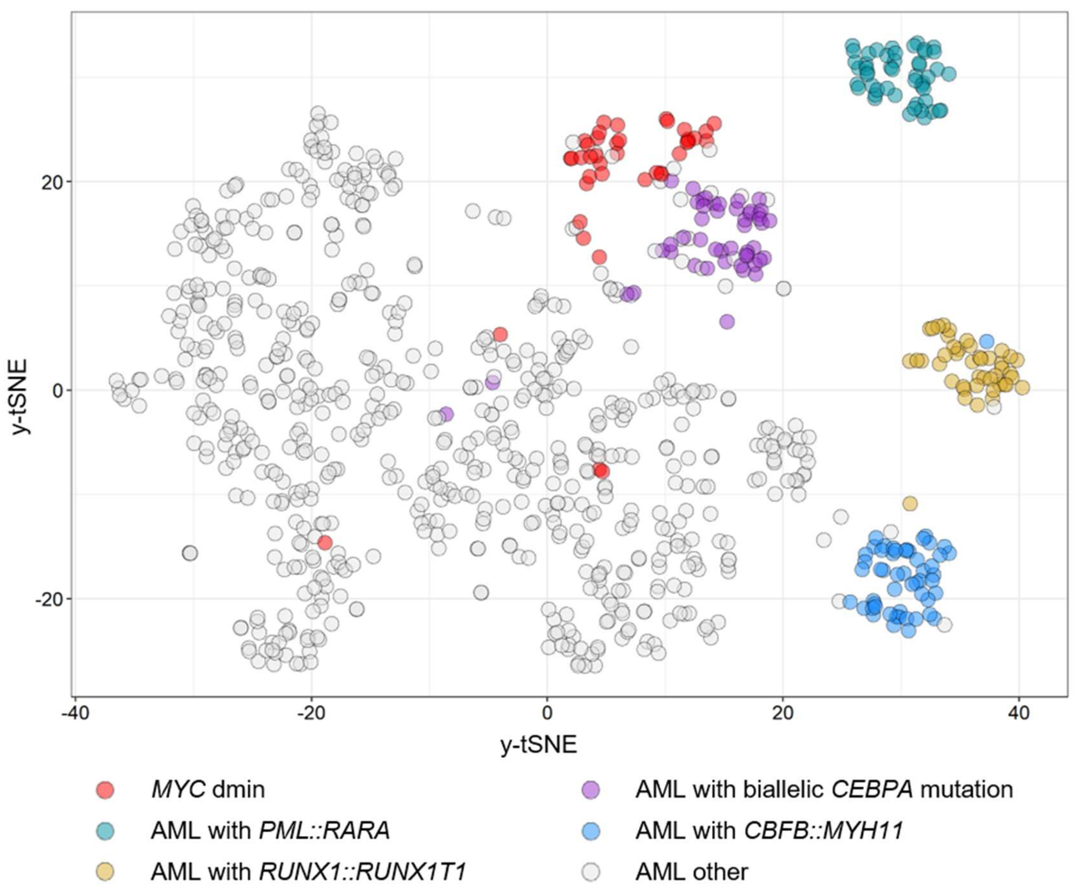

Supplementary Figure 5: Gene expression profiling of 40 *MYC* dmin cases and 830 AML cases of various subtypes shown in a two-dimensional t-distributed stochastic neighbor embedding (tSNE) plot based on the top 5% most variable genes (on the basis of median absolute deviation). Each dot visualizes one sample.

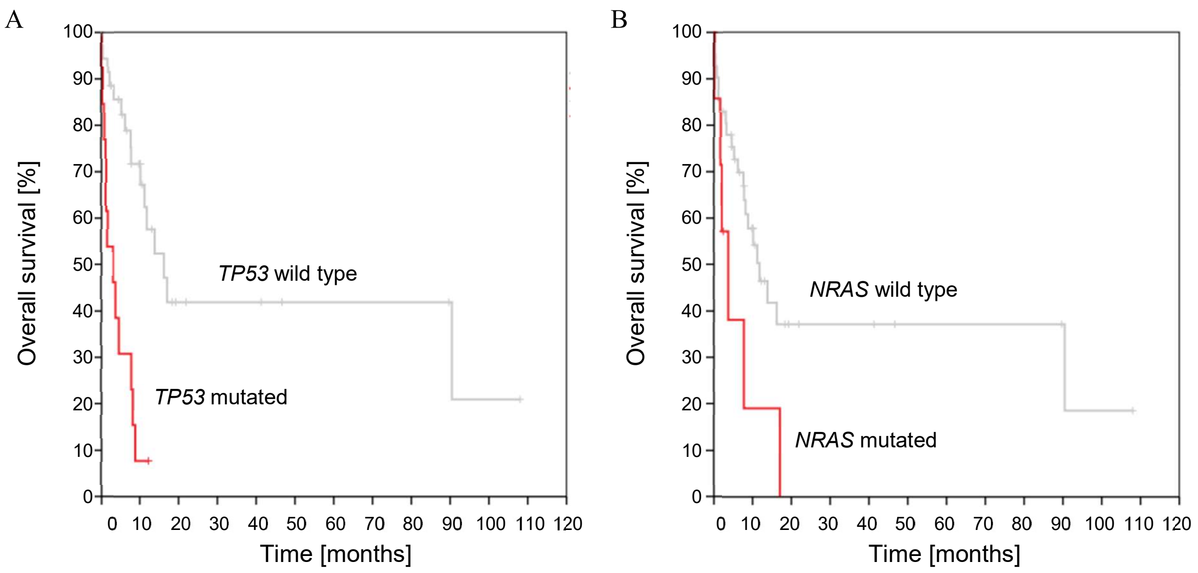

Supplementary Figure 6: (A) Overall survival of *TP53* mutated (red, n=13) vs. *TP53* wild type *MYC* dmin cases (grey, n=36) **(B)** Overall survival of *NRAS* mutated (red, n=8) vs. *NRAS* wild type *MYC* dmin cases (grey, n=41).

Supplementary Tables

Supplementary Table 1: Detailed information on 76 *MYC* dmin cases.

Supplementary Table 2: List of genes encoded within the common amplified region on Chr8q24.
